# The abundance and diversity of fruit flies and their parasitoids change with elevation in guava orchards in a tropical Andean forest of Peru, independent of seasonality

**DOI:** 10.1371/journal.pone.0250731

**Published:** 2021-04-26

**Authors:** Paolo Salazar-Mendoza, Ivan Peralta-Aragón, Ladislao Romero-Rivas, Jordano Salamanca, Cesar Rodriguez-Saona

**Affiliations:** 1 Departmento de Entomologia e Acarologia, Universidade de São Paulo, Escola Superior de Agricultura “Luiz de Queiroz”, Piracicaba, São Paulo, Brazil; 2 Escuela de Agronomía, Universidad Nacional Daniel Alcides Carrión, filial Oxapampa, Pasco, Peru; 3 Escuela de Ciencias Agrícolas, Pecuarias y del Medio Ambiente (ECAPMA), Universidad Nacional Abierta y a Distancia (UNAD), Bogotá, Colombia; 4 Department of Entomology, Rutgers University P.E. Marucci Center, Chatsworth, New Jersey, United States of America; United States Department of Agriculture, UNITED STATES

## Abstract

Lower elevations are generally thought to contain a greater abundance and diversity of insect communities and their natural enemies than higher elevations. It is less clear, however, how changes in seasons influence this pattern. We conducted a 2-year study (2013‒2014) in guava orchards located in a tropical Andean forest of Peru to investigate differences in fruit flies (Diptera: Tephritidae) and their parasitoid communities at two elevations and over two seasons. Fruit fly traps were installed, monitored, and guava fruits were sampled from eight orchards at low (800–950 m above sea level) and high (1,700–1,900 m above sea level) elevations and during the dry and rainy seasons. At each orchard, adult fruit fly trap captures and emergence of fruit flies and their parasitoids from guava fruit were quantified to determine their abundance and species composition. There was a greater abundance and species richness of fruit flies captured in traps at lower elevations, as well as higher abundance and species evenness of fruit flies that emerged from fruit, indicating that lower elevations are associated with larger fruit fly populations. The abundance, species richness and diversity of parasitoids were also greater at lower elevations. Consequently, guava fruit infestation and fruit fly parasitism rates were also greater at lower elevations. Seasonality also influenced fruit fly populations with a greater number of flies emerging from guava fruit and more fruit infested in the rainy season. However, seasonality had no effect on parasitoid population parameters or rate of parasitism, nor did it interact with elevation as an influence of populations of fruit flies or their parasitoids in guava orchards. This study highlights the importance of examining both elevation and seasonality for a better understanding of the population dynamics of fruit flies and their parasitoids in tropical agroecosystems.

## Introduction

Environmental conditions associated with higher elevation are known to influence insect communities [1]. For example, it is widely accepted that there is a decrease in the abundance and diversity of many insect groups at higher elevations, including beetles (Coleoptera) [2–5], moths and butterflies (Lepidoptera) [6–8], wasps (Hymenoptera) [9,10], and flies (Diptera) [1,11,12]. In addition, insect communities change according to season. Although seasonal fluctuations in tropical regions are not as drastic as in other latitudes, climatic changes due to seasonality can also affect insect abundance and diversity in the tropics [7], particularly because of seasonal differences in precipitation [11,13]. For example, Achumi et al. [11] showed a positive correlation of *Drosophila* fly population density with rainfall. Still, this is not true for all dipterans because several species of flies respond differently to seasonal variation [1]. While there have been studies on the effects of elevation and seasonality on insect communities, research on whether elevation interacts with seasonality to alter the communities of insect herbivores and their natural enemies is still largely missing.

Several fruit fly species (Diptera: Tephritidae) are important pests of fruit crops, causing major economic losses to growers worldwide. In South America, *Anastrepha* spp. and the Mediterranean fruit fly *Ceratitis capitata* (Wiedemann) are the most common fruit flies attacking commercial and non-commercial fruits [14] and are considered pests of quarantine importance in the fruit-growing regions [15,16]. *Anastrepha* spp. are native to the American continent and mainly restricted to tropical regions, with several species considered to be of economic importance [16]. In contrast, *C*. *capitata* is a species native to Africa that is adapted to diverse climates, including many tropical and temperate regions [17]. The abundance and diversity of these fruit fly species in tropical regions have been favored by a high diversity of hosts and climate conditions [15], which are also influenced by elevation [18,19] and seasonality [20,21]. Therefore, the abundance and species diversity of fruit flies are expected to change at different elevations and seasons.

Insect parasitoids belonging to the families Braconidae and Figitidae commonly attack fruit fly larvae in the American continent [22]. Of special note is the genus *Doryctobracon* (Hymenoptera: Braconidae), which is widely distributed in tropical regions [23,24]. These koinobiont parasitoids use several chemical cues to recognize their fruit fly host [25,26], then lay their eggs inside the larvae and emerge as adults in the pupal stage [27]. Cooler, and often misty, conditions at higher elevations can reduce the parasitoids’ searching capacity for fruit fly hosts and, thus, limit their efficacy [28,29]. Moreover, parasitoid species richness and parasitism rates generally decrease with increasing elevation [30,31]. However, the community-level effects of elevation on parasitoids are still unclear. For example, a group of parasitoid species of frugivorous drosophilid flies (named “ananassae”) was more abundant and achieved higher parasitism levels at lower elevations, whereas the abundance and level of parasitism of another group of species (“immigrans”) were not affected by elevation [32].

The montane forest ecosystem of the Peruvian Andes is recognized worldwide as rich in biological diversity [33–35]. The Oxapampa Province in Pasco (Peru) is located on the eastern slope of the Andean mountains (central tropical forest), which is characterized by montane forests composed of distinct ecological zones along different elevations. Guava (*Psidium guajava* L., Myrtaceae) is a fruit native to the American tropics [36] and commonly grown in this region, with great commercial potential in the fresh and processed markets [37]. Guava trees grow at elevations of up to 2,000 m above sea level [38] and bear fruit two times a year in these regions [39]. Guava fruit are suitable hosts for several fruit fly species, including the guava fruit fly (*Anastrepha striata* Schiner), the South American fruit fly (*Anastrepha fraterculus* (Wiedemann)), the West Indian fruit fly (*Anastrepha obliqua* (Macquart)), and *C*. *capitata* [14,40,41]. A variety of larval parasitoids are commonly associated with these fruit fly species [42,43].

In this study, we hypothesized that variation in elevation and seasonality is associated with differences in the community composition of fruit flies and their parasitoids in guava orchards in a tropical Andean forest in Oxapampa, Peru. Although we expected communities of fruit flies and their parasitoids at low elevations to be more complex than those at higher elevations, how these communities respond to variation in seasonality at different elevations is unknown. Our objective was to investigate the effects of elevation and seasonality on various community-level parameters, e.g., abundance and diversity (i.e., richness, evenness, Simpson’s index, and Shannon’s index), of fruit flies and their parasitoids in guava orchards. We used trapping and fruit collection to monitor tephritid populations during two fruiting seasons (dry and rainy seasons) at two elevations over two years (2013‒2014). Data from the sampled fruit were also used to assess the levels of fruit infestation by, and parasitism of, fruit flies in guava orchards.

## Material and methods

### Study area

**Fig 1:**
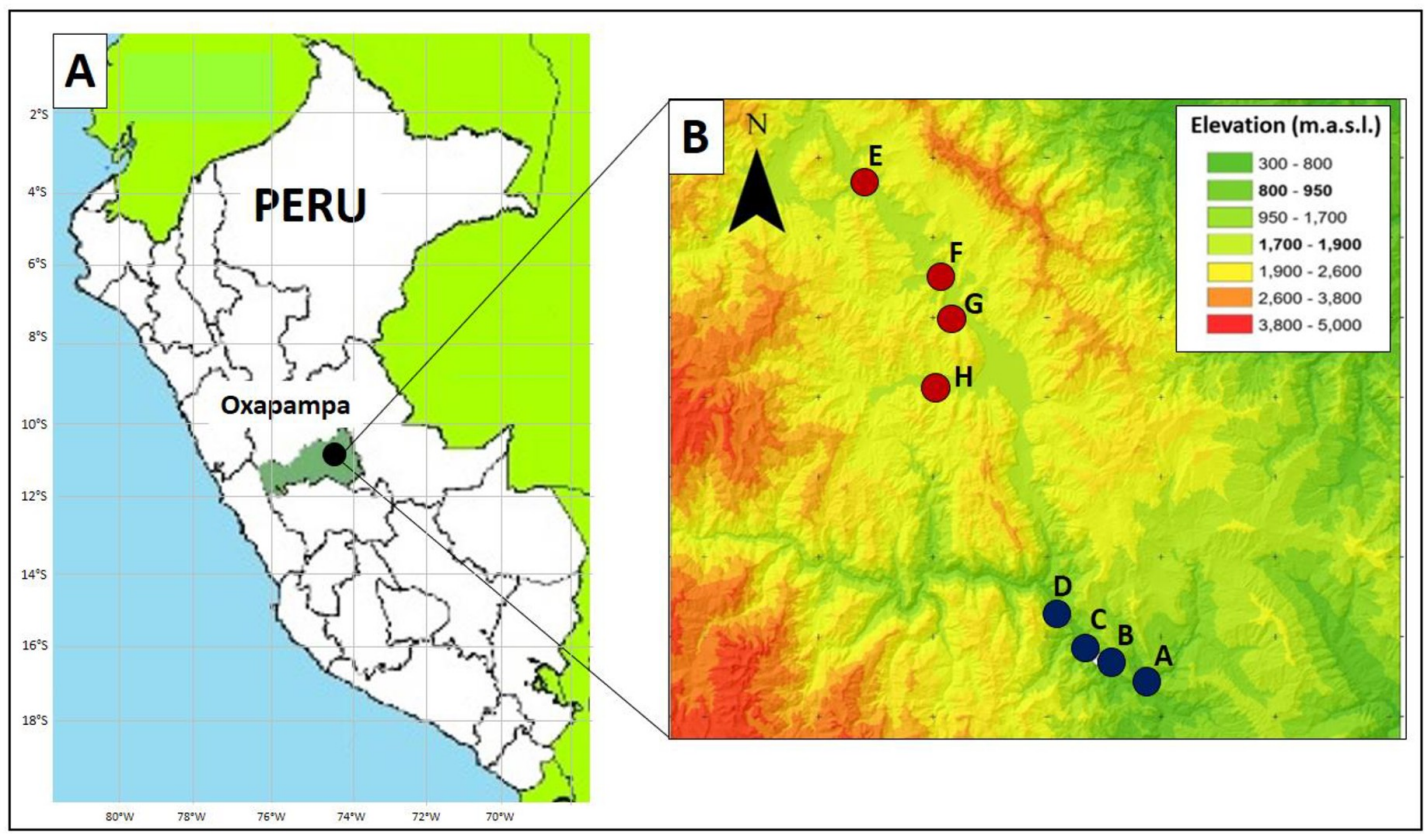
Location of sampling sites. A: Map of the study site (Oxapampa, eastern Pasco, Peru). B: Close-up of the eight farm locations: four farms (A‒D) were at the low elevation (800–950 m above sea level) and four farms (E‒H) were at the high elevation (1,700–1,900 m above sea level). See S1 Table for details on geographical coordinates and elevation for each farm. m.a.s.l. = meters above sea level. (source: QGIS 3.10.14, https://www.qgis.org, Accessed 29 Jan 2021).

This 2-year study (2013‒2014) was carried out in eight guava, *P*. *guajava* var. ‘white,’ orchards (1‒2 ha each) located in eight different commercial farms (one orchard per farm) in Oxapampa (eastern Pasco, Peru) (Fig 1). The study was conducted under the consent of the participating farmers, and no samples were collected in protected locations or included protected species; therefore, no specific permits were required. This region has two distinct seasons based on the amount of rainfall: a dry “winter” season (May to October) and a rainy “summer” season (November to April). Although in this region guava fruit ripens during both seasons, peak harvesting periods are February–March for the rainy season crop and September for the dry season crop. Guava orchards were divided into two groups based on their altitude [S1 Table]. Four orchards were selected from a low elevation (800–950 m above sea level) (Fig 1B, S1 Table), which is characterized as a tropical premontane forest [44], and located at the transition between the Amazonian forest and forest of the Andean slopes. The other four orchards were selected from a high elevation (1,700–1,900 m above sea level; i.e., two times higher than the low elevation) (Fig 1B, S1 Table), which is characterized as a tropical lower montane forest [44]. Thus, the study was designed as a 2 × 2 factorial, with two elevation gradients (low and high) and two seasons (dry and rainy).

Within each altitudinal group, the distance between orchards was at least 3 km. At all sites, guava fruits were an available resource to fruit flies during the two seasonal periods (dry and rainy) and at both altitudinal locations (S2 Table). Moreover, the four orchards within each altitudinal group exhibited similar patterns of other available fruit species. Besides guava, other hosts for fruit flies at the low elevation were mainly sweet orange (*Citrus sinensis* L.), mandarin (*Citrus reticulata* L.), tangelo (*Citrus paradisi* Macf. x *Citrus reticulata* Blanco), and other citrus crops. In the high elevation orchards, guava trees were commonly grown near coffee (*Coffea arabica* L.) and loquat (*Eriobotrya japonica* Lindl.). These species produce fruit at different times of the year and are common hosts of fruit flies in our study site (S2 Table). All guava orchards used in this study were surrounded by native forest and farmers followed conventional agronomic practices without use of insecticides for fruit fly control. In these orchards, guava is grown mainly for local consumption and the production of guava jelly.

### Trapping

In each orchard, we placed a single McPhail trap (Great Lakes IPM, Inc., Vestaburg, MI, USA) baited with protein hydrolysate (Buminal^®^; Aventis Cropscience Peru S.A, Lima, Peru) (20 mL/250 mL water). These McPhail traps and baits are commonly used to capture females and males of several fruit fly species in control programs [45]. Five grams of borax (sodium tetraborate; Fausto Piaggio S.A, Callao, Peru) were added to the water-bait mixture as a preservative. Traps were placed on a branch of a guava tree, 2.5–3 m above the ground, in an open area (i.e., not touching the tree canopy) to increase their visibility. The traps were serviced every seven days and the bait replaced. The trapped fruit flies were placed in vials (50 ml) containing 70% alcohol and taken to the laboratory for taxonomic identification. In each farm, traps were monitored for 20 consecutive weeks from November to April during the dry seasons, and for 20 consecutive weeks from May to October during the rainy seasons, totaling eighty trap samples per elevation per season.

### Fruit sampling

In each season and year, we collected eight fruit samples [1 sample = 5 fruits (~ 0.27 kg)] in 4 different weeks (depending on availability) from each orchard, i.e., 32 samples/orchard, n = 8 orchards, for total of 256 samples for all orchards. Each year, fruit samples were taken throughout seven months during the dry (August–October) and rainy (January–April) seasons. Mature, ripe fruit were collected from the guava trees to increase the probability of finding fruit flies (healthy and parasitized) [46]. Fruits were collected randomly from different guava trees depending on their availability and placed in paper bags that were sealed to prevent fly larvae from escaping. Bags were kept inside plastic coolers and taken to the Universidad Nacional Daniel Alcides Carrión (UNDAC) (Oxapampa, Peru) for processing. In the laboratory, samples were weighed and placed in Styrofoam boxes (38 × 20 × 20 cm) with a 4 cm layer of sterilized and moistened sand in the bottom to allow larvae emerging from the fruit to pupae. Fruit fly larvae found in paper bags were added to the box containing fruit from the same sample. Every four days, the sand covering the bottom of the containers was sieved to collect the pupae. Three weeks after field collections, the boxes were inspected and fruits were dissected to assess the presence (dead or alive) of fruit fly larvae or pupae. Live larvae and pupae were subsequently transferred to plastic cups (500 ml) with sterilized and moistened sand and covered with a fine mesh on the top. Daily, for up to two months, plastic cups were checked for adult fruit fly emergence or emergence of larval parasitoids. Laboratory conditions were 22 ± 3°C, 65 ± 10% relative humidity, and 12:12 light:dark; these conditions were comparable to those where the fruits were sampled.

### Species identification

Fruit flies collected in traps and those emerged from fruit samples were identified to species using Tephritidae keys [15,47]. Fruit fly species identification was confirmed by the Fruit Flies and Phytosanitary Projects [Moscas de la Fruta y Proyectos Fitosanitarios (SMFPF)] at the National Service of Agrarian Health [Servicio Nacional de Sanidad Agraria (SENASA)] in Lima, Peru. Fruit fly parasitoid species were identified using Braconidae and Figitidae keys [24,48]. Fruit fly parasitoid species identification was confirmed by Angelica Penteado-Dias (Federal University of São Carlos, SP, Brazil) and Valmir Costa (Agronomic Institute of Campinas, Brazil). Voucher specimens of fruit flies and parasitoid species were deposited at the Entomology Museum of the UNDAC.

### Weather conditions

Weather conditions (temperature and rainfall) from January to December of 2013 and 2014 were obtained from a weather station located in each of the two elevations. For the low and high elevations, weather conditions were obtained from the Cerro La Sal Ecolodge, Oxapampa (10.8322°S 75.2902°W, 832 m above sea level) and the Oxapampa Agrarian Agency (Agencia Agraria Oxapampa, Oxapampa, Peru; 10.5788°S 75.4033°W, 1,809 m above sea level), respectively.

### Data analyses

All statistical analyses were conducted using R ver. 3.3.1 [49]. For each farm, season, and year, the total abundance, number of species (species richness), the relative abundance of each species (species evenness), Simpson’s index, and Shannon’s index were determined for fruit flies and their parasitoids using the software PAST 4.02 [50]. These data were first checked for normality using the Shapiro–Wilk test [51] and for homoscedasticity using the Levene’s test (‘car’ package in R). The effects of season (dry and rainy) and elevation (low and high), and the interaction between them, on the abundance and diversity (richness, evenness, Simpson’s index, and Shannon’s index) of fruit flies and their parasitoids were tested by 2-way analysis of variance (ANOVA). Prior to analyses, data were averaged for all sampling dates within each season and elevation to obtain a single (mean) value for each orchard (experimental unit). If needed, data were transformed before ANOVA using ln(x + 0.05) to meet assumptions of normality.

Fruit infestation levels by fruit flies were calculated according to Aluja et al. [52]: fruit infestation level per mass = number of pupae from a fruit sample/sample weight (kg). Percent parasitism was calculated according to Steck et al. [53]: percent parasitism = a/(a + b) × 100, where a = number of recovered parasitoid and b = number of emerged fruit flies in each sample. The effects of the season and elevation, and the interaction between them, on fruit infestation and parasitism levels were tested by 2-way ANOVA, using the orchards as experimental units. Before ANOVA, data on percent parasitism were arcsine square-root transformed.

## Results

### Fruit flies in traps

**Fig 2:**
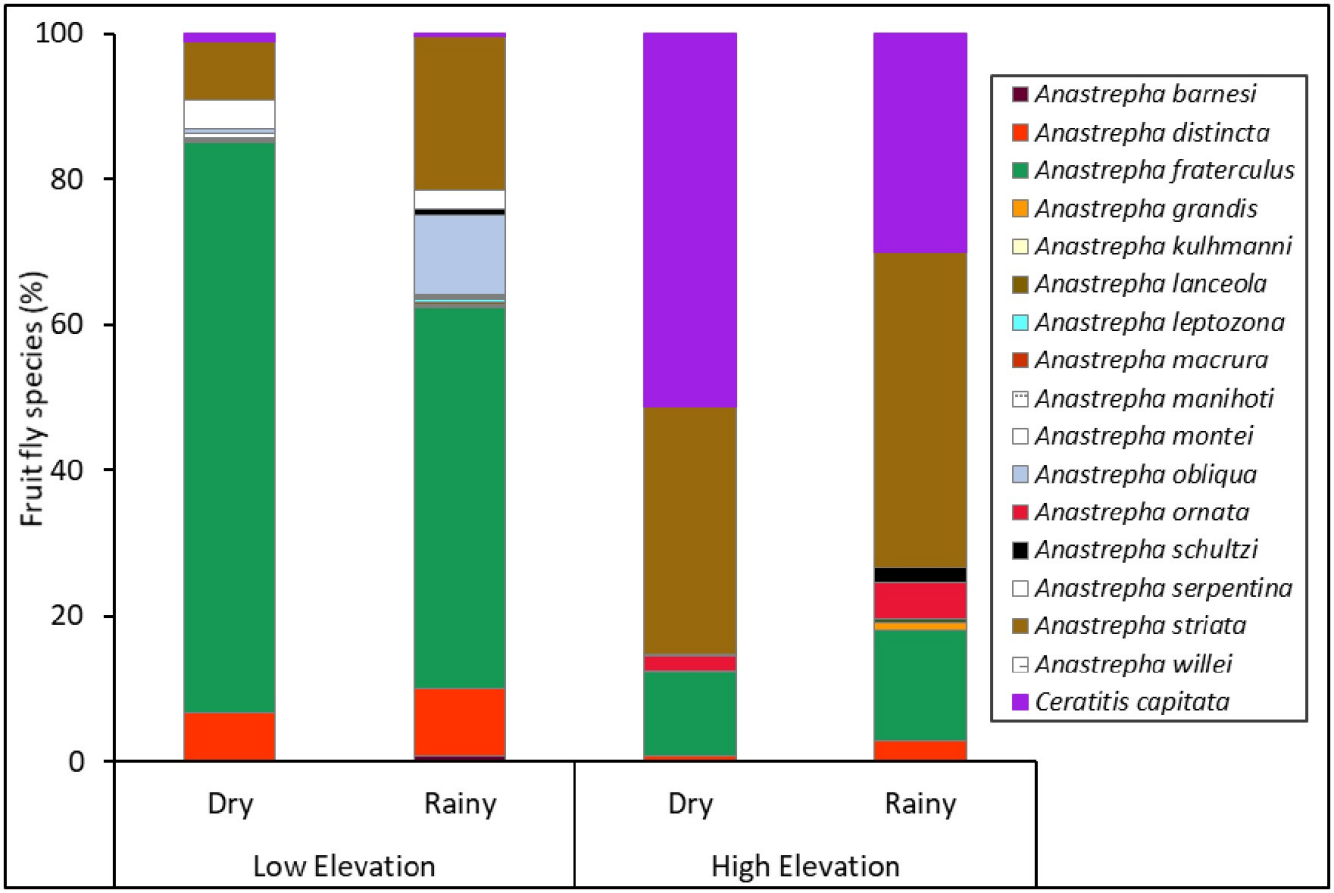
Fruit fly species composition in traps. Percent of fruit fly species captured in traps placed in guava orchards at different elevations (low and high) and seasons (dry and rainy) from 2013‒2014.

**Fig 3:**
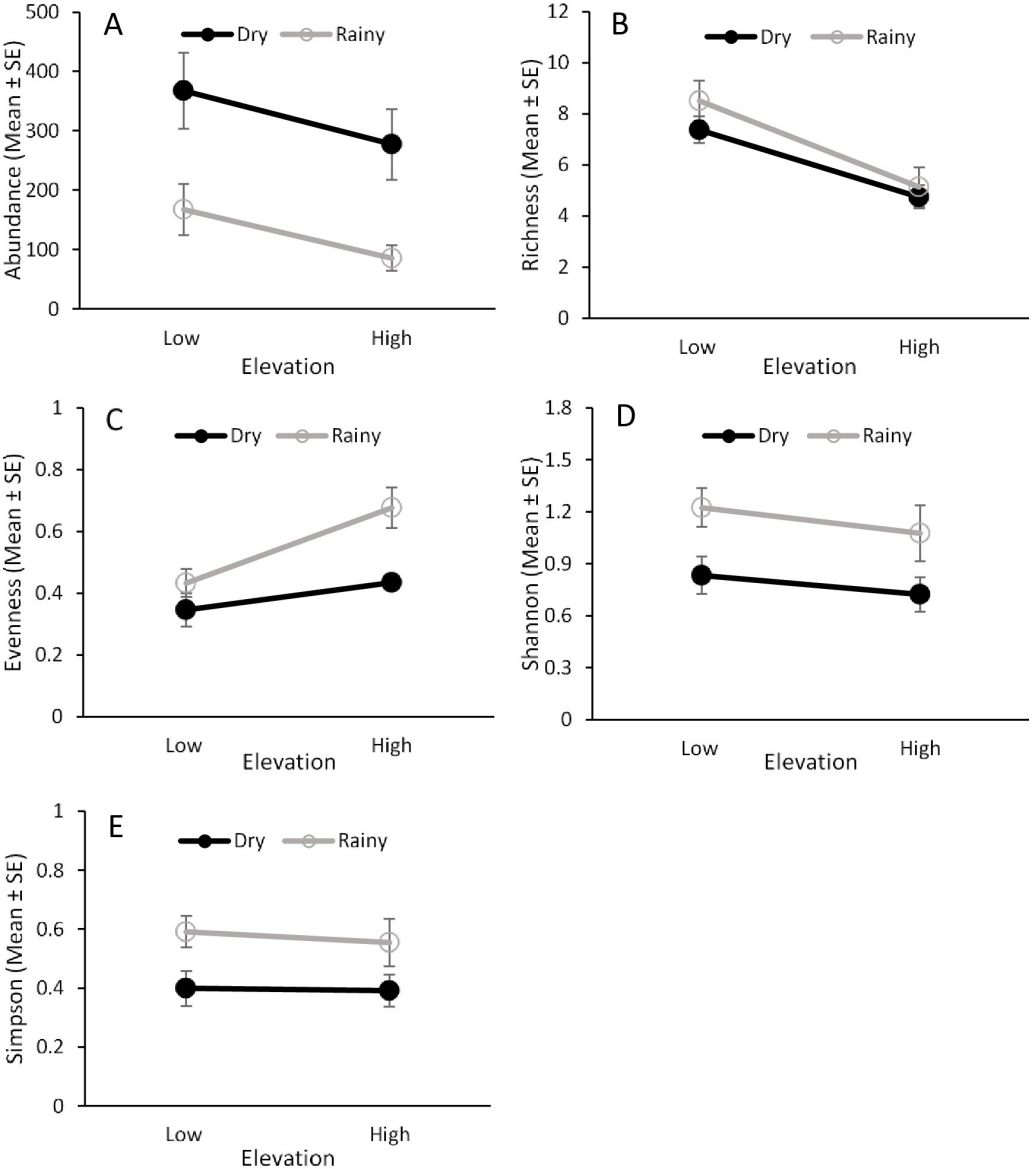
Fruit fly abundance and species diversity in traps. Effects of elevation (low and high) and seasonality (dry and rainy) on the abundance (A), species richness (B), species evenness (C), and Shannon’s (D) and Simpson’s (E) indices for fruit flies captured in traps placed in guava orchards from 2013‒2014.

*Ceratitis capitata* and sixteen *Anastrepha* species were captured in traps during the study (Fig 2, S3 Table). Elevation and seasonality had a significant effect on the abundance and evenness of fruit flies in traps, but there was no interaction effect between these two factors (Table 1). Higher numbers of fruit flies were captured at the lower elevation and during the dry season (Fig 3A), while evenness was higher at the high elevation and in the rainy season (Fig 3C).

**Table 1:** Results of 2-way analysis of variance for the effects of elevation and season, and the interaction between these two factors, on fruit flies and their parasitoids in guava orchards.

Variable	Source	Fruit flies in traps		Fruit flies in fruits		Fruit fly parasitoids	
		*F*^a^	*P-value*^b^	*F*^a^	*P-value*^b^	*F*^a^	*P-value*^b^
Abundance	Elevation	4.35	**0.04**	7.17	**0.01**	7.28	**0.01**
	Season	18.33	**<0.001**	20.17	**<0.001**	3.08	0.08
	Elevation × Season	0.79	0.38	0.004	0.95	0.13	0.71
Richness	Elevation	20.97	**<0.001**	1.32	0.25	7.65	**<0.01**
	Season	1.3	0.26	1.32	0.25	3.03	0.09
	Elevation × Season	0.32	0.57	1.32	0.25	0.51	0.47
Evenness	Elevation	11.55	**<0.01**	20.67	**<0.001**	1.57	0.22
	Season	11.27	**<0.01**	0	0.98	1.87	0.18
	Elevation × Season	2.49	0.12	2.85	0.1	3.21	0.08
Shannon’s index	Elevation	1.09	0.3	1.14	0.29	4.86	**0.03**
	Season	9.09	**<0.001**	0.07	0.78	1.33	0.25
	Elevation × Season	0.02	0.87	0.61	0.43	1.21	0.27
Simpson’s index	Elevation	0.45	0.5	0.55	0.46	4.46	**0.03**
	Season	2.07	0.16	0.22	0.63	1.25	0.25
	Elevation × Season	0.15	0.69	0.18	0.66	1.07	0.3

^a^Error: df = 1,28.

^b^Numbers in bold indicate significant effects at α = 0.05.

Fruit fly species richness was influenced by elevation but not by seasonality or the interaction between them (Table 1). Higher species richness was found at the low elevation (Fig 3B). Shannon’s diversity index was influenced by seasonality but not by elevation or the interaction between them (Table 1). This index showed greater diversity in the rainy season than in the dry season (Fig 3D). No effect of elevation, seasonality, or their interaction was found for the Simpson’s index (Fig 3E, Table 1).

### Fruit flies in fruits

**Fig 4:**
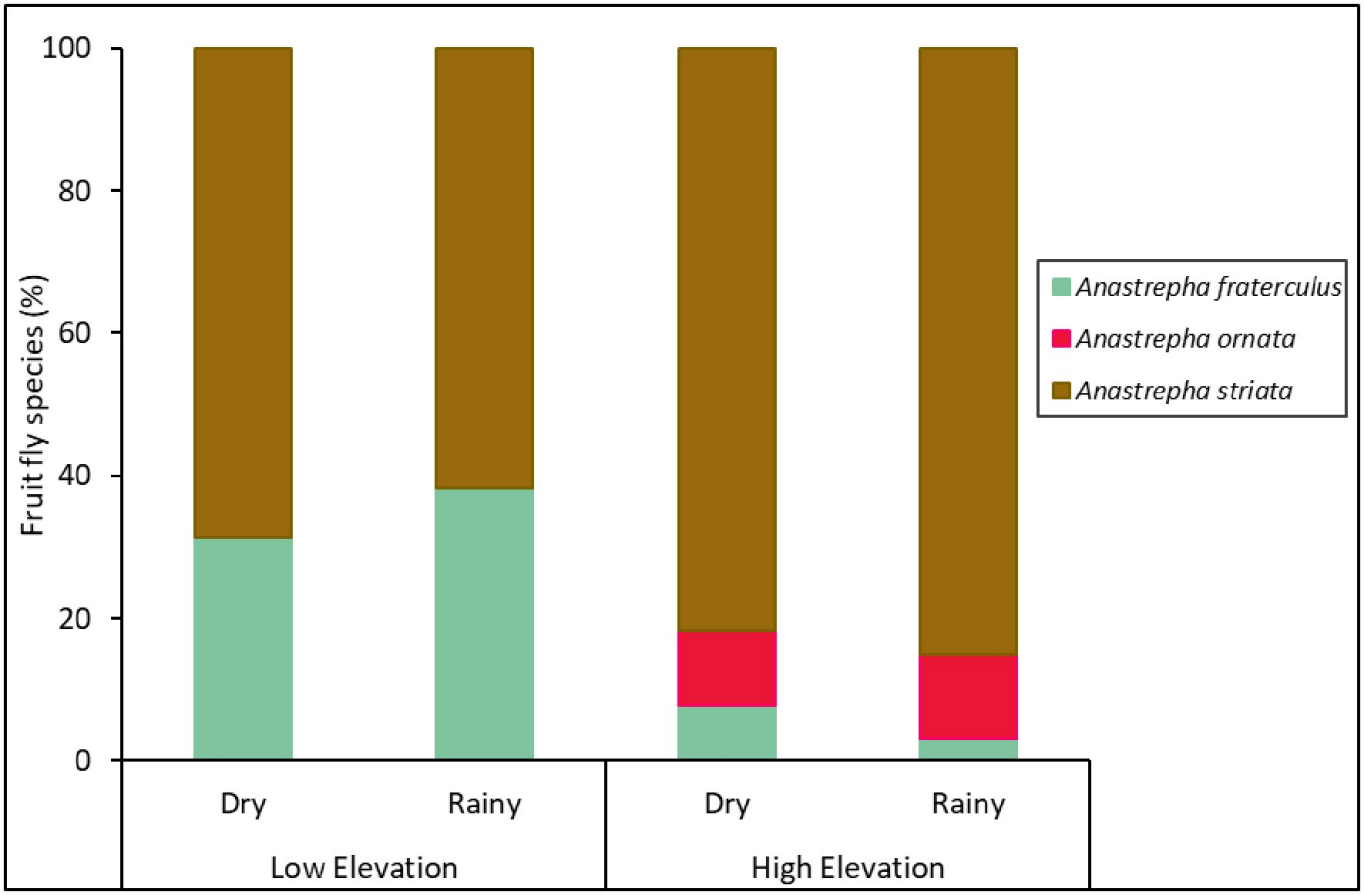
Fruit fly species composition in fruit. Percentage of fruit fly species that emerged in guava fruit collected at different elevations (low and high) and seasons (dry and rainy) from 2013‒2014.

**Fig 5:**
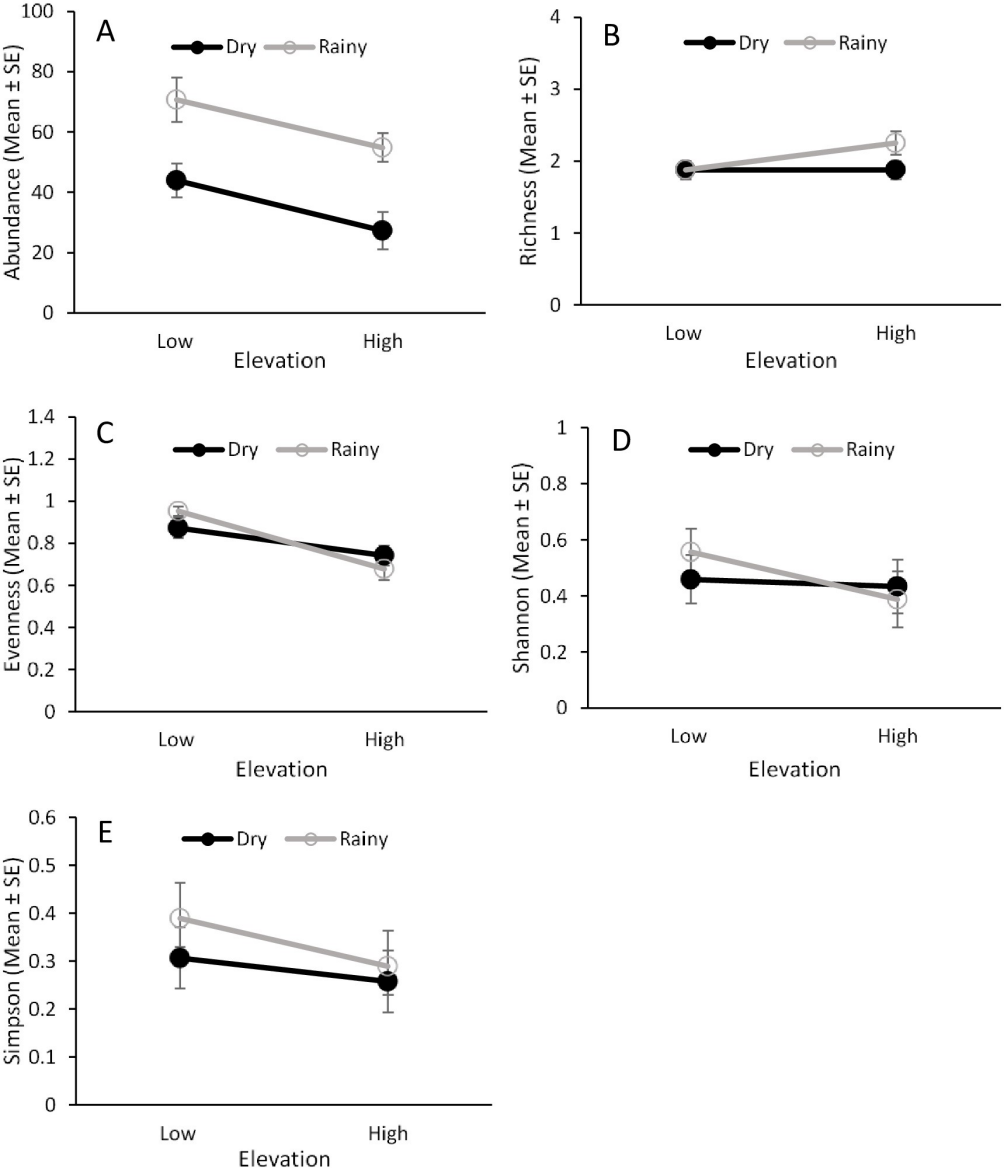
Fruit fly abundance and species diversity in fruit. Effects of elevation (low and high) and seasonality (dry and rainy) on the abundance (A), species richness (B), species evenness (C), and Shannon’s (D) and Simpson’s (E) indices for fruit flies that emerged from guava fruit from 2013‒2014.

Only three *Anastrepha* species (*A*. *fraterculus*, *Anastrepha ornata* Aldrich, and *A*. *striata*) emerged from guava fruit (Fig 4, S4 Table). Elevation and seasonality separately had a significant effect on the abundance of fruit fly species emerging from guava fruit, but there was no interaction between these two factors (Table 1). More fruit flies emerged from fruit collected at low elevations and during the rainy season (Fig 5A).

Analysis of species evenness found that there was an effect of elevation, but not an effect of seasonality or an interaction between them (Table 1), with higher species evenness at lower elevations (Fig 5C). There was no effect of elevation, seasonality, or their interaction on species richness (Fig 5B) and the Simpson’s and Shannon’s diversity indices (Fig 5D and 5E, Table 1).

### Fruit fly parasitoids

**Fig 6:**
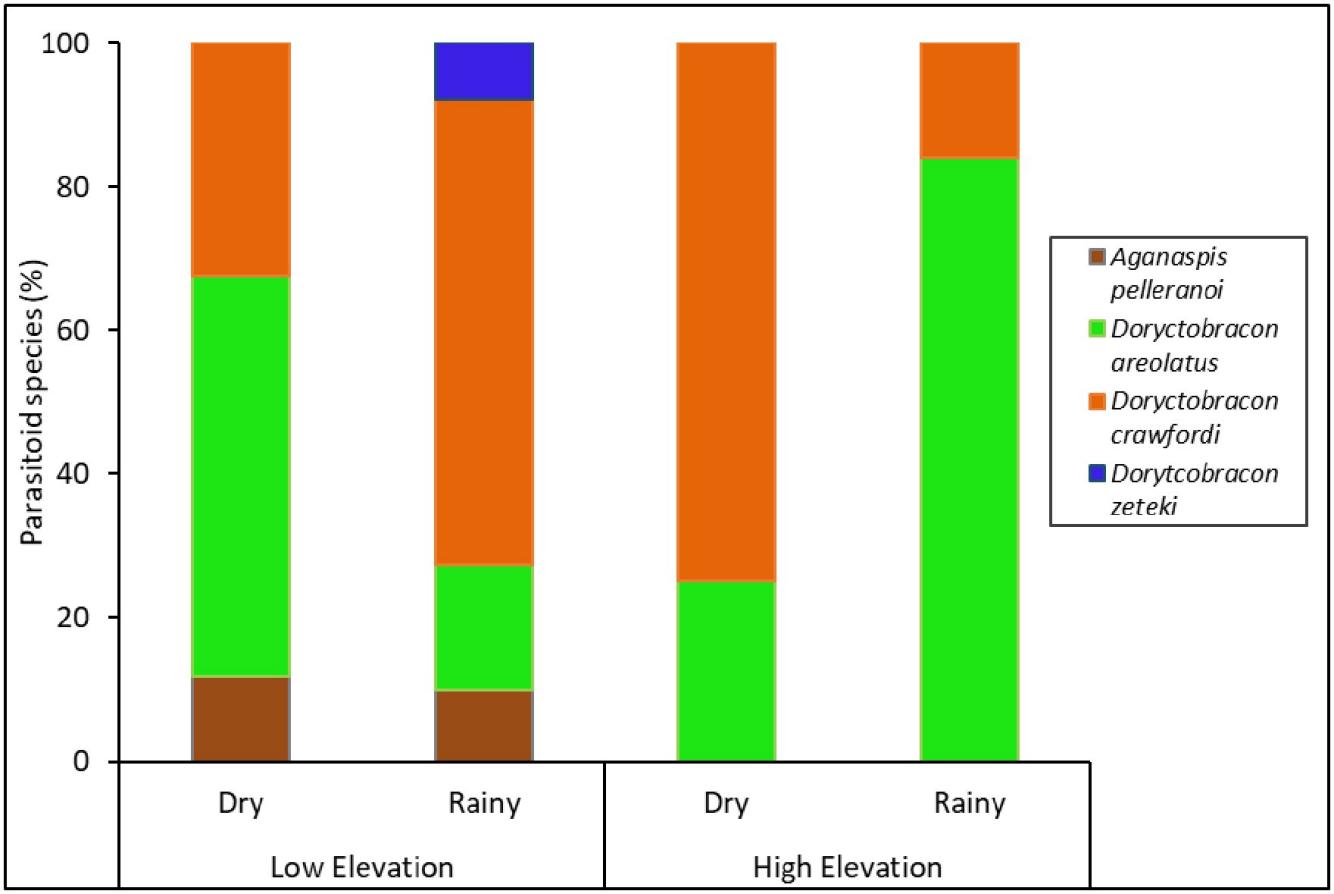
Fruit fly parasitoid species composition in fruit. Percentage of fruit fly larval parasitoids that emerged from guava fruit collected at different elevations (low and high) and seasons (dry and rainy) from 2013‒2014.

**Fig 7:**
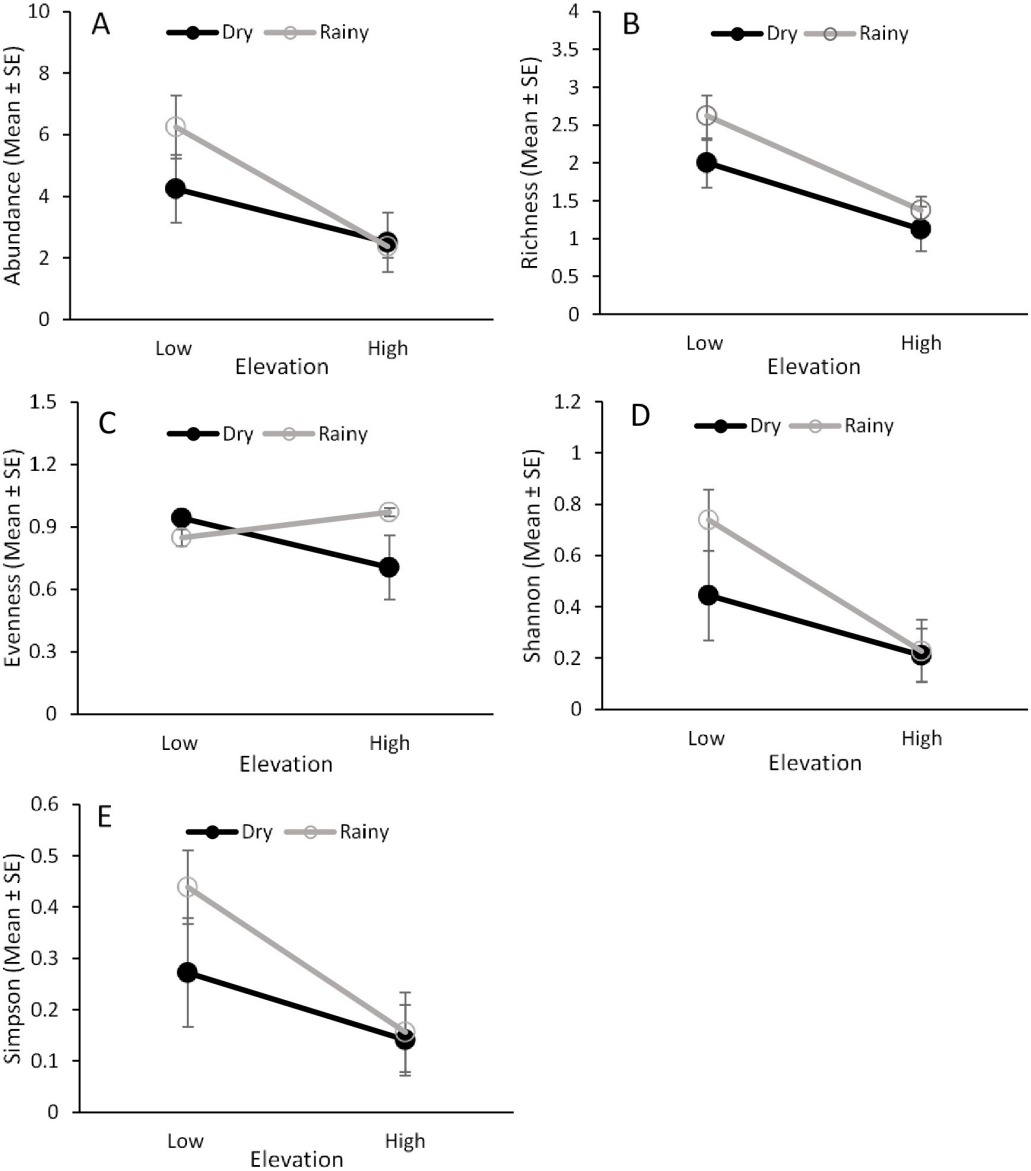
Fruit fly parasitoid abundance and species diversity in fruit. Effects of elevation (low and high) and seasonality (dry and rainy) on the abundance (A), species richness (B), species evenness (C), and Shannon’s (D) and Simpson’s (E) indices for fruit fly parasitoids that emerged from guava fruit from 2013‒2014.

Four fruit fly larval parasitoids, *Aganaspis pelleranoi* (Bréthes) (Hymenoptera: Figitidae), *Doryctobracon areolatus* (Szépligeti) (Hymenoptera: Braconidae), *Doryctobracon crawfordi* (Viereck) (Hymenoptera: Braconidae), and *Doryctobracon zeteki* (Muesebeck) (Hymenoptera: Braconidae), emerged from individuals collected from guava fruit (Fig 6, S5 Table). The abundance and species diversity (richness and Simpson’s and Shannon’s indices), but not species evenness, of these parasitoids were significantly affected by elevation (Fig 7, Table 1). The lower elevation had greater abundance and higher parasitoid species diversity than the higher elevation (Fig 7). In fact, the parasitoids *A*. *pelleranoi* and *D*. *zeteki* were found only at the lower elevation (Fig 6, S5 Table). Neither seasonality nor the interaction between elevation and seasonality affected parasitoid abundance and the diversity indices.

### Infestation and parasitism levels

**Fig 8:**
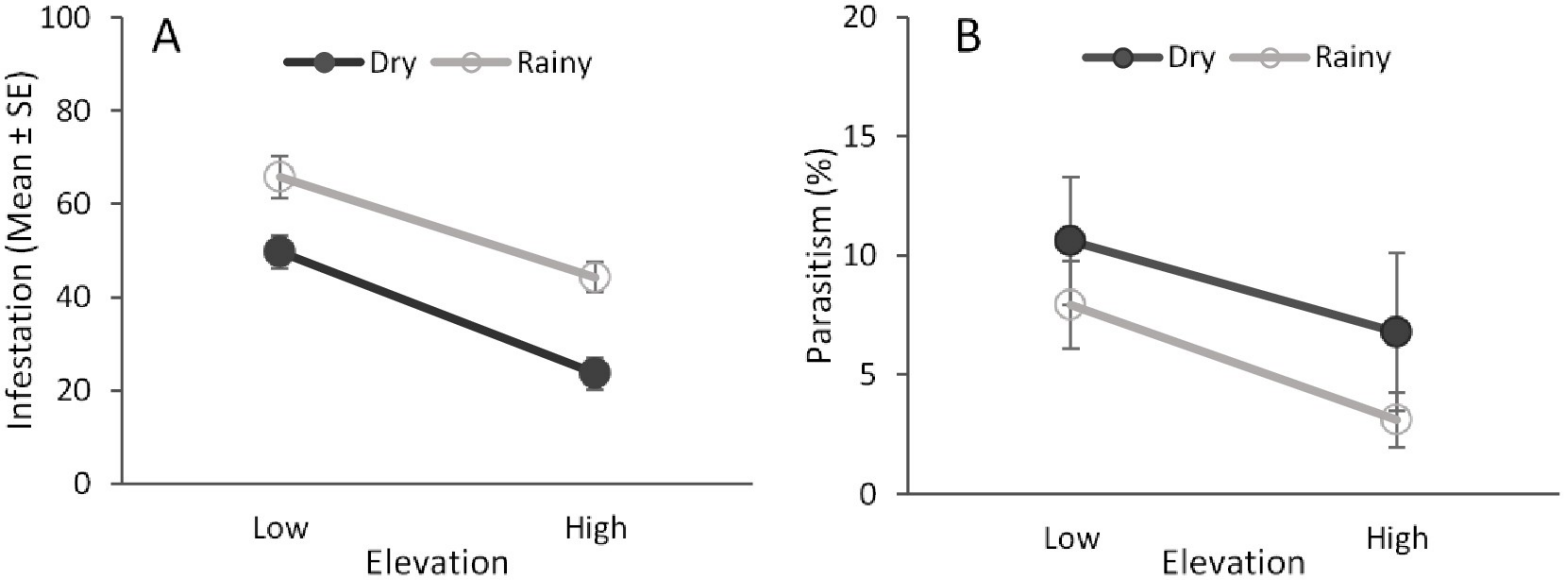
Fruit infestation and parasitism rates. Effects of elevation and seasonality on the levels of fruit infestation by fruit flies (A) and fruit fly parasitism (B) of guava fruit collected at different elevations (low and high) and seasons (dry and rainy).

Elevation and seasonality, but not their interaction, had a significant effect on fruit fly infestation level of guava fruit (elevation: *F* = 46.14; df = 1,252, *P* < 0.001; season: *F* = 32.93; df = 1,252; *P* < 0.001; elevation × season: *F* = 2.48; df = 1,252; *P* = 0.11). Fruit infestation was higher at the low elevation and during the rainy season (Fig 8A).

Parasitism rates were also significantly higher at the low elevation (*F* = 4.6; df = 1,252, *P* = 0.03) (Fig 8B). There were no effects of seasonality (*F* = 0.07; df = 1,252, *P* = 0.78) or the interaction between elevation and seasonality (*F* = 1.76; df = 1,252, *P* = 0.18) on parasitism rates (Fig 8B).

### Weather conditions

**Fig 9:**
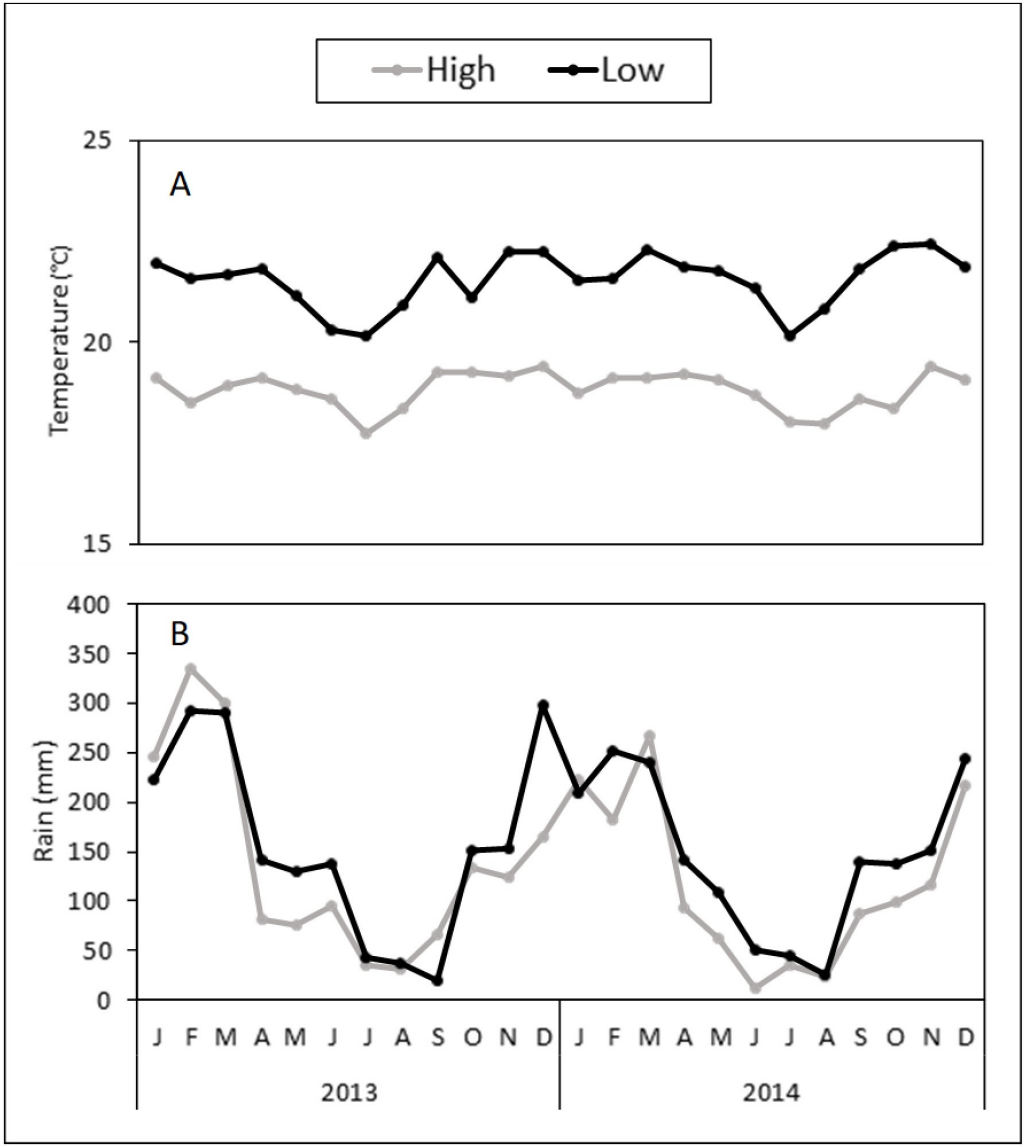
Weather conditions at study sites. Monthly temperature (A) and rainfall (B) at different elevations (low and high) and seasons (dry and rainy) in Oxapampa, Peru, from 2013‒2014. The dry “winter” season corresponds from May to October, while the rainy “summer” season corresponds from November to April.

Over the 2 years of this study (2013‒2014), the monthly mean temperatures were relatively constant throughout the years (≤3°C difference between temperature maximum and minimum). Temperature was 14.4% higher at the low elevation (mean ± SE = 21.5°C ± 0.1) than at the high elevation (18.8°C ± 0.1) (Fig 9). However, there was little variation in temperature between the rainy (20.5°C ± 0.3) and dry (19.9°C ± 0.3) seasons (Fig 9).

Total rainfall clearly defined the two seasons in both years: the amount of rain was 2.8 times higher in the rainy (4,973 mm) than in the dry (1,777 mm) season (Fig 9). Overall, rainfall was greater at the low (3,656 mm) than the high (3,095 mm) elevation (Fig 9).

## Discussion

Insect communities are expected to differ over a variety of elevations and seasons; however, whether they act independently or interact to modify the communities of fruit flies and their parasitoids in tropical regions remains poorly understood. In guava orchards located in a tropical Andean forest, we showed that (1) at lower elevations, there was a greater abundance and species richness of fruit flies captured in traps as well as higher abundance and species evenness of fruit flies that emerged from guava fruit. (2) The abundance and species diversity (richness and Shannon and Simpson’s indices) of fruit fly parasitoids were also higher at lower elevations in larvae recovered from guava. (3) As a result, guava fruit infestation and fruit fly parasitism rates were greater at lower elevations. (4) Fruit fly abundance also changed with seasonality, which resulted in greater fruit infestation in the rainy season; however, seasonality had no effect on any parasitoid population parameters or on parasitism rate. (5) Seasonality did not interact with elevation to influence communities of fruit flies or their parasitoids in guava orchards.

Elevation influences various environmental parameters, such as temperature, precipitation, atmospheric pressure, and solar radiation [54,55], which can in turn affect the survival and reproduction of insects as well as their interactions with other organisms. In general, environmental conditions associated with high elevations result not only in a decrease in overall insect abundance and diversity but also in the structural complexity of the insects’ habitat [30,56]. In this study, temperature and rainfall were different in the high compared to low elevations, such that both parameters were greater at low elevations. More fruit flies were captured in traps at low elevations and their diversity was greater, likely in response to the higher temperatures and precipitation, whereas evenness was reduced because one species (*A*. *fraterculus*) was dominant at low elevations. Similar to our findings, Berrones-Morales et al. [19] found increased fruit fly species richness but less species evenness at the lowest elevations across different ecosystems over a Neotropical transitional region in Mexico. Also, Geurts et al. [57] reported the over-dominance of *Bactrocera invadens* Drew adults captured in traps at low elevations in Tanzania. Altogether, previous results and ours indicate that lower elevations are generally associated with greater fruit fly abundance and species richness, in which species evenness is reduced due to the dominance of a few species.

*Anastrepha* is considered the most diverse genera of fruit flies in American tropical and subtropical regions, with more than 300 identified species [16,58], many of which often damage fruit crops in the Peruvian Andean forest regions [58,59, S2 Table]. Interestingly, in this study, most of the fruit fly species captured in traps in guava orchards were not found attacking the guava fruits because only 3 out of 16 *Anastrepha* species present in the region infested this fruit [21,60,61]. Adults of both *A*. *striata* and *A*. *fraterculus* emerged from guava fruits at the low and high elevations, whereas *A*. *ornata* adults emerged from guava fruits only at the high elevation. Similar to the trapping data, we found greater abundance of fruit flies emerging from guava fruits at low elevations, which resulted in more fruit being infested at low elevations. Unlike the trapping data, however, measures of species evenness of fruit flies emerging from guava fruit were greater at low elevations due to the dominance of *A*. *striata* at high elevations. These results contradict those by Birke and Aluja [40] who found *A*. *striata* to be more dominant than *A*. *fraterculus* at lower elevations in guava in Veracruz, México, suggesting geographical differences in the response of fruit fly species to elevation. It should be noted that no fruit fly emergence was observed in ~42% of pupae under our laboratory conditions and we do not know if this detrimental effect was similar across species, which could have introduced a bias. Additional laboratory studies will be needed to determine if the pupal survival of fruit fly species differs under different environmental conditions.

Several factors could be responsible for the observed increase in abundance and diversity of fruit flies at lower elevations. One factor could be a reduction in habitat structure (e.g., size and diversity) at high elevations [62], which could affect the availability of certain fruits to particular fruit fly species [30]. Although fruit crop types differed between the two elevations in our study, hosts were available all year round at both elevations (S2 Table). Moreover, the density of fruit on guava trees was similar in both low and high elevations (P.S.-M. and I.P.-A., personal observation) and, thus, not likely to explain the observed differences in fruit fly abundance and diversity. Environmental conditions at high elevations can also affect the host plants, such as their morphology, phenology, size, physiology, chemistry, and spatial configuration [30,63]. For example, lower levels of nutrients available to plants at high elevations could negatively impact fruit fly performance [64,65]. Also, several defensive plant secondary metabolites, such as alkaloids, coumarins, phenolics, and terpenes, usually vary along elevational gradients [65–68], with a trend toward a decrease in chemical defenses at higher elevations due to reduced selection pressure by herbivores. In addition, elevation is associated with changes in fruit fly physiology (e.g. flight activity, diapause intensity, and regulation of energy reserves) [69,70], which can alter their survival, growth and development. Largely, fruit fly developmental times are inversely proportional to temperature [71,72]; thus, fruit fly development should be shorter at lower elevations. Also, Duyck et al. [73] showed an increase in *Ceratitis rosa* Karsch lifespan with higher elevations, which could slow their reproduction. Moreover, the results here suggest the co-existence of various fruit fly species in guava orchards, which could be mediated in part by differences in their host ranges and adaptations to different environmental conditions [28,74]. For instance, *A*. *fraterculus* is a polyphagous fruit fly particularly abundant in guava at low elevations, possibly due to a greater availability of alternative hosts (S2 Table). In contrast, *A*. *striata* is more associated with guava and other myrtaceous fruits [75], and their abundance was greater at higher elevations. These non-mutually exclusive external and internal factors could have contributed to the differential susceptibility of guava fruits to fruit flies at different elevations, and further research is needed to determine their role in shaping fruit fly communities in this Andean tropical region.

In general, we found that fruit flies and their parasitoid communities had similar responses to elevation [76]. This study showed increased fruit fly parasitoid abundance and species diversity (richness, Simpson’s index, and Shannon’s index) at low elevations, likely due to the greater abundance and diversity of their fruit fly hosts [77]. As a result, more infested fruit was parasitized at low elevations. Other studies have also shown that the abundance of parasitoid species, levels of parasitism, and parasitoid species richness often decrease with increasing elevation [30,31]. Cooler temperatures (and misty conditions) associated with higher elevations could diminish the capacity of some parasitoid species to locate their hosts and consequently reduce their efficacy in parasitizing them [28,29]. Several studies also showed a decrease in parasitoid functional efficiency at temperatures below an optimum [78–80]. However, the effects of elevation on fruit fly parasitoids can also be species specific. For example, Sivinski et al. [28] reported higher *D*. *areolatus* emergence from guava and “jobo” (*Spondias mombin* L.) fruits at low elevations, whereas emergence of *D*. *crawfordi* from sour orange (*Citrus aurantium* L.) was greater at high elevations in Veracruz, México. In contrast, we found similar numbers of both *D*. *areolatus* and *D*. *crawfordi* emerging from guava at low and high elevations, whereas *A*. *pelleranoi* and *D*. *zeteki* were collected only from the guavas collected at low elevations. These dissimilarities in the response of specific fruit fly parasitoid species to elevation could be due in part to differences in host availability at different geographic locations. It should be pointed out that our study likely underestimated the true parasitism rates in the orchards because fruits infested with fruit fly larvae were removed from the field, thus reducing the time the larvae were exposed to parasitism.

In addition to elevation, environmental conditions due to seasonality can affect insect communities and those of their parasitoids [8]. Unlike temperate regions where extreme winter temperatures often regulate fluctuations in insect communities, in tropical regions, temperatures remain within an ideal range for insect development throughout the year [81]. In tropical regions, seasonality is instead strongly marked by precipitation. Our data show a clear difference between the dry and rainy seasons in precipitation but not in temperature, with greater amounts of rainfall in the rainy season. Likely due to these differences, the abundance of fruit flies captured in traps was higher in the dry season. Similarly, other studies conducted in tropical regions found higher fruit fly populations during the dry season [82–84]. Seasons with overabundant rainfall could reduce fruit fly immature survival [85]. In contrast, Aluja et al. [18] captured more fruit flies in traps at ~300 mm of rainfall (comparable to levels seen in our rainy season), but the authors did not find a clear relationship between precipitation and population density. It is possible that fruit fly attraction to our “wet” traps increased in the dry season when water may be limiting; thus, further studies are needed to investigate the attraction of fruit flies to these traps during the dry and wet seasons. However, fruit fly species evenness and diversity (Shannon’s index) from traps were higher in the rainy season. These differences in fruit fly abundance and diversity between seasons could be due to the availability of fruits. For instance, seasonal shifts in the availability of fruit hosts could result in higher fruit fly abundance or an increase of rare species depending on the season. In fact, Celedonio-Hurtado et al. [21] suggest that the principal factor driving seasonal fluctuations of fruit fly population density is fruit availability. In the tropics, fruit fly seasonality is often linked to hosts that may serve as a bridge allowing populations of fruit flies to persist at times of the year when the main hosts are not available [40,86], resulting in host shifts depending on the season.

In contrast to trap captures, the abundance of fruit flies that emerged from guava fruit as well as the level of fruit infestation were higher in the rainy season. As indicated above, in tropical regions, there are variations in fruit availability to fruit flies depending on seasonality [18,21,87]. However, even when hosts are available in both dry and rainy seasons such as guava, it is possible that their quantity and quality (i.e., amount of secondary metabolites and nitrogen) differ along seasonal climatic conditions. For instance, dry conditions can lead to reduced photosynthesis and growth in plants [88,89], which could result in lower fruit fly populations in guava. Importantly, seasonality did not influence the effects of elevation on any of the fruit fly population-level parameters either based on trap counts or fruit emergence, indicating that these two factors acted independently in shaping fruit fly communities.

A reduction in herbivore populations due to seasonal environmental conditions can negatively affect the third trophic level [89,90]. For instance, precipitation negatively affected the parasitism of fruit flies in Veracruz, México [91], in southeastern Brazil [92], and in the Argentinean Yungas [87]. In contrast, we found that fruit fly parasitoid abundance, species diversity, and parasitism rates were not influenced by precipitation. This finding agrees with results by Wong et al. [93] who found no relationship between seasonality and fruit fly parasitism rate. A likely explanation is that our study used only one host (guava), whereas the previous studies used a succession of several fruits throughout the season. In fact, the availability of fruit and fruit fly species composition within these fruits are key determinants of parasitoid communities [87], favoring the maintenance of their populations even in periods of guava scarcity. Regardless, our study clearly shows that elevation more strongly influences fruit fly parasitoid communities in guava orchards in Oxapampa, Peru, than seasonality (dry versus rainy).

## Conclusions

We conclude that elevation is an important factor influencing the communities of fruit flies and their parasitoids in guava orchards in a tropical Andean forest of Peru. In general, there was a negative relationship between fruit fly and fruit fly parasitoid abundance and species diversity with increasing elevation. Although certain population-level parameters of fruit flies, but not those of their parasitoids, were also influenced by seasonality, this effect was independent of elevation. Likely, changes in environmental conditions, nutritional and defensive chemistry in fruits, and the availability of others hosts contributed to our findings, and additional studies are needed to disentangle these possible mechanisms. The findings presented in this study for guava, fruit flies, and their parasitoids in the tropics are particularly relevant in light of the potential effects of climate change on tri-trophic interactions in agroecosystems.

## Supporting information

S1 Table Geographic information of orchards. This dataset contains the geographic location of the eight guava orchards sampled during 2013‒2014 with the corresponding coordinates and elevation.
(DOCX)

S2 Table Fruiting phenology of host plants in guava orchards. This dataset contains the fruiting phenology of host plants in guava orchards, and the fruit flies associated with them, at the low and high and season during the dry and rainy season from 2013–2014.
(DOCX)

S3 Table Fruit fly species in traps. This dataset contains the total number and percentage of each fruit fly species collected in traps at the low and high elevations and during the dry and rainy seasons from 2013‒2014.
(DOCX)

S4 Table Fruit fly species that emerged from fruit. This dataset contains the total number and percentage of fruit fly species that emerged from guava fruit collected at the low and high elevations and during the dry and rainy seasons from 2013‒2014.
(DOCX)

S5 Table Fruit fly parasitoids that emerged from fruit. This dataset contains the total number and percentage of fruit fly parasitoids that emerged from guava fruit collected at the low and high elevations and during the dry and rainy seasons from 2013‒2014.
(DOCX)

S1 Data (XLSX)
