# Supplementary material for: The abundance and diversity of fruit flies and their parasitoids change with elevation in guava orchards in a tropical Andean forest of Peru, independent of seasonality
Source: PLoS One. 2021 Apr 26;16(4):e0250731. doi: 10.1371/journal.pone.0250731 (PMC8075242; doi:10.1371/journal.pone.0250731)
Supplement: S1 Table — This dataset contains the geographic location of the eight guava orchards sampled during 2013‒2014 with the corresponding coordinates and elevation. (DOCX) [file pone.0250731.s001.docx]

**S1 Table.** Geographical location and elevation of guava orchards sampled during 2013‒2014.

| **Code** | **Orchards** | **Geographical Location**  **(°S, °W)** | **Elevation (m.a.s.l.)^1^** |
| --- | --- | --- | --- |
| A | Puente Paucartambo | 10.8286, 75.2925 | 847.23 |
| B | Sogormo | 10.8166, 75.3238 | 865.9 |
| C | Playapampa | 10.8091, 75.3363 | 918.7 |
| D | Churumazu | 10.7869, 75.3516 | 935.6 |
| E | Huancabamba | 10.4463, 75.5008 | 1772.6 |
| F | Gramazu | 10.5158, 75.4488 | 1792.1 |
| G | Tsachopen | 10.5461, 75.4344 | 1809.1 |
| H | San Martin | 10.6027, 75.3850 | 1850.5 |
| ^1^m.a.s.l. = meters above sea level. | | | |
