# Supplementary material for: The abundance and diversity of fruit flies and their parasitoids change with elevation in guava orchards in a tropical Andean forest of Peru, independent of seasonality
Source: PLoS One. 2021 Apr 26;16(4):e0250731. doi: 10.1371/journal.pone.0250731 (PMC8075242; doi:10.1371/journal.pone.0250731)
Supplement: S2 Table — This dataset contains the fruiting phenology of host plants in guava orchards, and the fruit flies associated with them, at the low and high and season during the dry and rainy season from 2013–2014. (DOCX) [file pone.0250731.s002.docx]

**S2 Table.** Fruiting phenology of host plants in guava orchards, and the fruit flies associated with them, at different elevations and season in Oxapampa, Pasco, Peru (January to December 2013‒2014).^1^

| **Elevation** | **Host plant** | | **Fruit fly species** | **Seasons^2^** | | | | | | | | | | | |
| --- | --- | --- | --- | --- | --- | --- | --- | --- | --- | --- | --- | --- | --- | --- | --- |
|  |  |  |  | **Rainy** | | | | **Dry** | | | | | | **Rainy** | |
|  | **Common name** | **Species name** |  | **J** | **F** | **M** | **A** | **M** | **J** | **J** | **A** | **S** | **O** | **N** | **D** |
| High | Coffee | *Coffea arabica* L. | *Ceratitis capitata* (Wiedemann) |  |  |  |  |  |  |  |  |  |  |  |  |
|  | Guava | *Psidium guajava* L. | *Anastrepha fraterculus* (Wiedemann)*, Anastrepha ornata* Aldrich*, Anastrepha striata* Schiner |  |  |  |  |  |  |  |  |  |  |  |  |
|  | Loquat | *Eriobotrya japonica* Lindl. | *A. fraterculus* |  |  |  |  |  |  |  |  |  |  |  |  |
|  | Rangpur lime | *Citrus limonia* Osbeck | *A. fraterculus* |  |  |  |  |  |  |  |  |  |  |  |  |
| Low | Abiu | *Pouteria caimito* (Ruiz & Pav.) | *A. fraterculus, Anastrepha serpentina* (Wiedemann) |  |  |  |  |  |  |  |  |  |  |  |  |
|  | Guava | *Psidium guajava* L. | *A. fraterculus, A. striata* |  |  |  |  |  |  |  |  |  |  |  |  |
|  | Ice cream-bean | *Inga edulis* Mart. | *Anastrepha distincta* Greene*, A. fraterculus* |  |  |  |  |  |  |  |  |  |  |  |  |
|  | Mandarin | *Citrus reticulata* L. | *A. fraterculus* |  |  |  |  |  |  |  |  |  |  |  |  |
|  | Mango | *Mangifera indica* L. | *A. fraterculus, Anastrepha obliqua* (Macquart) |  |  |  |  |  |  |  |  |  |  |  |  |
|  | Sweet orange | *Citrus sinensis* L. | *A. fraterculus* |  |  |  |  |  |  |  |  |  |  |  |  |
|  | Rough lemon | *Citrus jambhiri* Lush | *A. fraterculus* |  |  |  |  |  |  |  |  |  |  |  |  |
|  | Rangpur lime | *Citrus limonia* Osbeck | *A. fraterculus* |  |  |  |  |  |  |  |  |  |  |  |  |
|  | Tangelo | *C. reticulata* Blanco x *C. paradisi* Macf. | *A. fraterculus* |  |  |  |  |  |  |  |  |  |  |  |  |
| ^1^Peralta-Aragón & Salazar-Mendoza, unpublished data.  ^2^Black, gray, and while shading indicates high, low, and no availability of fruits, respectively. | | | | | | | | | | | | | | | |
