# Supplementary material for: The abundance and diversity of fruit flies and their parasitoids change with elevation in guava orchards in a tropical Andean forest of Peru, independent of seasonality
Source: PLoS One. 2021 Apr 26;16(4):e0250731. doi: 10.1371/journal.pone.0250731 (PMC8075242; doi:10.1371/journal.pone.0250731)
Supplement: S3 Table — This dataset contains the total number and percentage of each fruit fly species collected in traps at the low and high elevations and during the dry and rainy seasons from 2013‒2014. (DOCX) [file pone.0250731.s003.docx]

**S3 Table.** Number and percentage of fruit fly species collected in traps at different elevations and seasons in 2013‒2014.

| **Species** | Low Elevation | | | | High Elevation | | | | Total | |
| --- | --- | --- | --- | --- | --- | --- | --- | --- | --- | --- |
|  | Dry | | Rainy | | Dry | | Rainy | |  |  |
|  | N | % | N | % | N | % | N | % | N | % |
| *Anastrepha barnesi* | 1 | 0.03 | 11 | 0.82 | 0 | 0 | 0 | 0 | 12 | 0.17 |
| *Anastrepha distincta* | 199 | 6.78 | 124 | 9.23 | 18 | 0.81 | 20 | 2.89 | 361 | 5.02 |
| *Anastrepha fraterculus* | 2301 | 78.35 | 703 | 52.35 | 255 | 11.50 | 104 | 15.05 | 3363 | 46.79 |
| *Anastrepha grandis* | 3 | 0.10 | 3 | 0.22 | 5 | 0.23 | 8 | 1.16 | 19 | 0.26 |
| *Anastrepha kulhmanni* | 9 | 0.31 | 3 | 0.22 | 0 | 0 | 0 | 0 | 12 | 0.17 |
| *Anastrepha lanceola* | 0 | 0 | 3 | 0.22 | 0 | 0 | 4 | 0.58 | 7 | 0.10 |
| *Anastrepha leptozona* | 4 | 0.14 | 7 | 0.52 | 0 | 0 | 0 | 0 | 11 | 0.15 |
| *Anastrepha macrura* | 1 | 0.03 | 2 | 0.15 | 0 | 0 | 0 | 0 | 3 | 0.04 |
| *Anastrepha manihoti* | 0 | 0 | 3 | 0.22 | 0 | 0 | 0 | 0 | 3 | 0.04 |
| *Anastrepha montei* | 16 | 0.54 | 3 | 0.22 | 0 | 0 | 0 | 0 | 19 | 0.26 |
| *Anastrepha obliqua* | 18 | 0.61 | 147 | 10.95 | 0 | 0 | 0 | 0 | 165 | 2.30 |
| *Anastrepha ornata* | 0 | 0 | 0 | 0 | 45 | 2.03 | 34 | 4.92 | 79 | 1.10 |
| *Anastrepha schultzi* | 1 | 0.03 | 10 | 0.74 | 5 | 0.23 | 14 | 2.03 | 30 | 0.42 |
| *Anastrepha serpentina* | 116 | 3.95 | 37 | 2.76 | 0 | 0 | 0 | 0 | 153 | 2.13 |
| *Anastrepha striata* | 235 | 8.00 | 282 | 21.00 | 754 | 34.01 | 299 | 43.27 | 1570 | 21.84 |
| *Anastrepha willei* | 1 | 0.03 | 2 | 0.15 | 0 | 0 | 0 | 0 | 3 | 0.04 |
| *Ceratitis capitata* | 32 | 1.09 | 3 | 0.22 | 1135 | 51.20 | 208 | 30.10 | 1378 | 19.17 |
| Total | 2937 |  | 1343 |  | 2217 |  | 691 |  | 7188 |  |
