# Supplementary material for: The abundance and diversity of fruit flies and their parasitoids change with elevation in guava orchards in a tropical Andean forest of Peru, independent of seasonality
Source: PLoS One. 2021 Apr 26;16(4):e0250731. doi: 10.1371/journal.pone.0250731 (PMC8075242; doi:10.1371/journal.pone.0250731)
Supplement: S5 Table — This dataset contains the total number and percentage of fruit fly parasitoids that emerged from guava fruit collected at the low and high elevations and during the dry and rainy seasons from 2013‒2014. (DOCX) [file pone.0250731.s005.docx]

**S5 Table.** Number and percentage of fruit fly parasitoids that emerged from guava fruit collected at different elevations and seasons from 2013‒2014.

| **Species** | Low Elevation | | | | High Elevation | | | | Total | |
| --- | --- | --- | --- | --- | --- | --- | --- | --- | --- | --- |
|  | Dry | | Rainy | | Dry | | Rainy | |  |  |
|  | N | % | N | % | N | % | N | % | N | % |
| *Aganaspis pelleranoi* | 4 | 11.8 | 5 | 9.8 | 0 | 0 | 0 | 0 | 9 | 7.3 |
| *Doryctobracon areolatus* | 19 | 55.9 | 9 | 17.6 | 5 | 25 | 16 | 84 | 49 | 40 |
| *Doryctobracon crawfordi* | 11 | 32.3 | 33 | 64.8 | 15 | 75 | 3 | 16 | 62 | 50 |
| *Dorytcobracon zeteki* | 0 | 0 | 4 | 7.8 | 0 | 0 | 0 | 0 | 4 | 3.2 |
| Total | 34 |  | 51 |  | 20 |  | 19 |  | 124 |  |
